# Mapping ERβ Genomic Binding Sites Reveals Unique Genomic Features and Identifies EBF1 as an ERβ Interactor

**DOI:** 10.1371/journal.pone.0071355

**Published:** 2013-08-08

**Authors:** Thien P. Le, Miao Sun, Xin Luo, W. Lee Kraus, Geoffrey L. Greene

**Affiliations:** 1 Ben May Department for Cancer Research, University of Chicago, Chicago, Illinois, United States of America; 2 Green Center for Reproductive Biology Sciences, University of Texas Southwestern, Dallas, Texas, United States of America; Karolinska Institutet, Sweden

## Abstract

Considerable effort by numerous laboratories has resulted in an improved understanding of estrogen and SERM action mediated by the two estrogen receptors, ERα and ERβ. However, many of the targets for ERβ in cell physiology remain elusive. Here, the C4-12/Flag.ERβ cell line which stably expressed Flag.ERβ is used to study ERβ genomic functions without ERα interference. Mapping ERβ binding sites in these cells reveals ERβ unique distribution and motif enrichment patterns. Accompanying our mapping results, nascent RNA profiling is performed on cells at the same treatment time. The combined results allow the identification of ERβ target genes. Gene ontology analysis reveals that ERβ targets are enriched in differentiation, development and apoptosis. Concurrently, E2 treatment suppresses proliferation in these cells. Within ERβ binding sites, while the most prevalent binding motif is the canonical ERE, motifs of known ER interactors are also enriched in ERβ binding sites. Moreover, among enriched binding motifs are those of GFI, REST and EBF1, which are unique to ERβ binding sites in these cells. Further characterization confirms the association between EBF1 and the estrogen receptors, which favors the N-terminal region of the receptor. Furthermore, EBF1 negatively regulates ERs at the protein level. In summary, by studying ERβ genomic functions in our cell model, we confirm the anti-proliferative role of ERβ and discover the novel cross talk of ERβ with EBF1 which has various implications in normal physiology.

## Introduction

Estrogens regulate homeostasis, development and reproduction by exerting their effects on a number of tissues, including the mammary gland, the brain, the cardiovascular system, the liver, the musculoskeletal system, the intestines and the immune system. These effects are mediated by one or both of the two estrogen receptors, ERα and ERβ. Given the diverse roles that estrogens exert on physiology, many studies have been devoted to understanding ER biology in these tissues [1], [2]. The importance of ERα in the genesis, treatment and prevention of breast cancer prevention is also well recognized, which has resulted in improved management of ERα-positive breast cancers. While ERβ is also expressed in many breast tumors, its role remains elusive and controversial [3], [4]. In the prostate and colon, ERβ is considered the predominant ER subtype, with a potential role as a tumor suppressor [5], [6]. In the brain and the ovaries, distinct functions have been reported for both ERs [7]–[9].

As members of the nuclear receptor superfamily, ERα and ERβ contain several canonical functional domains, including the activation function 1 (AF1) at the N-terminus, followed by the DNA-binding domain (DBD), the hinge region, and ligand-binding domain (LBD), which also contains the activation function 2 (AF2) at the C-terminal end. Despite being encoded by different genes, ERα and ERβ share significant homology. The two DBD’s are almost identical (97% homology), which allows both ERs to recognize a consensus estrogen responsive element (ERE) (GGTCAnnnTGACC) with equal efficacy. The two LBD’s share approximately 60% of their amino acid sequences, resulting in overlapping as well as distinct ligand recognition. The N-terminal region has the least similarity between the two ERs, which explains much of their differential roles in regulating cell physiology [10]–[12]. This region has also been suggested to regulate the proteasome-mediated degradation of ERβ [13].

Because the two ERs regulate cell physiology predominantly at the transcriptional level, understanding their interactions with the genome is crucial to elucidating ER biology. Thanks to advances in DNA sequencing technologies, the global interaction between ERα and the genome has been extensively characterized. Genome-wide mapping of ERα binding events in ERα-positive MCF-7 breast cancer cells has provided significant information regarding global distribution, motif enrichment patterns and target genes, which confirm many known ERα activities, including regulation of proliferation [14]–[16].

While the roles of ERα in transcriptional regulation in response to ligand binding are well studied, much remains to be learned regarding transcriptional regulation mediated by ERβ [4], [17]. ERβ mechanistic studies have been limited by two major challenges. The first of these is the lack of immortalized cell lines expressing significant amounts of endogenous ERβ. Although ERβ expression has been reported in several established cell lines, these observations remain controversial [18]–[22]. The second challenge is the general lack of validated, specific antibodies to detect ERβ in cells, tissues and tissue/cell extracts [23]. As a consequence, no genome-wide analysis of endogenous ERβ action has been reported yet, to the best of our knowledge.

To circumvent these issues, ERβ genomic functions have been investigated in cell lines engineered to express this receptor exogenously [19], [24], [25]. In MCF7 breast cancer cells that overexpress recombinant ERβ, shared binding sites have been observed for both ERs. Binding sites unique to ERβ have also been detected [16], [19], [24]. Similarly, in U2OS osteosarcoma cells engineered to express ERα, ERβ or both ERs, the two ER subtypes have overlapping sets of target genes as well as distinct target genes [25], [26]. In addition, the interplay between these ERs at the genomic level has also been reported in these models [16], [19], [24], [26]–[30]. Although the genomic functions of ERβ in a physiologically relevant context remain to be determined, these studies provide significant insights into ERβ actions at the genomic level. However, ERβ genomic functions in the absence of ERα still require further investigation.

In order to study ERβ genomic functions in the absence of ERα interference, we used MCF-7/C4–12 cells, a derivative of MCF-7 cells that has lost ERα expression [31]. Using lentiviral infection, we generated C4–12/Flag.ERβ cells that stably express Flag-tagged ERβ. To identify true ERβ target genes, ERβ genomic binding sites were mapped by ChIP-seq analysis while global nascent RNA generated at the time of binding was also captured with a GRO-seq assay [32], [33], Using this approach, 3166 E2-mediated ERβ binding sites were identified and 342 were found differentially regulated by ERβ. Furthermore, a novel cross talk between ERβ and Early B-cell Factor 1 (EBF1) was also identified and characterized.

## Materials and Methods

### Cell Cultures

All the cells, including HEK293, HEK293T, MCF7/C4–12 [33], C4–12/Flag.ERβ (which were derived from MCF7/C4–12 as described below), were cultured and propagated in DMEM with 10% Fetal Bovine Serum (FBS) as previously described [31]. Prior to all experiments, all cells were cultured in DMEM with 5% charcoal-stripped Fetal Bovine Serum (SFBS) for 48 hours.

### Generation of the C4–12/Flag.ERβ Cell Line

HEK293T cells were transfected with the Flag-ERβ pCDH, VSV-G, and deltaR vectors on day 1 using Lipofectamine 2000 (Life Technologies). After overnight incubation, transfecting media was replaced with normal culturing media to induce the production of lentiviruses. On day 3, media with lentivirus were collected, filtered, and used to treat MCF7/C4–12 cells [33] in the presence of polybrene. On day 4, lentiviral media was removed, treated with bleach, and discarded; the infected cells were culture in normal culturing media until they needed to be further propagated. The DNA cassette being incorporated into the host genome carried a green fluorescent protein (GFP) that was used as a selective marker of positively infected cells. Infected cells with high GFP expression were sorted and further propagated. After two rounds of sorting, resulting cell line was referred to as the C4–12/Flag.ERβ, in which the expressions of GFP and Flag-ERβ were detectable up to 50 passages.

### Chromatin Immunoprecipitation

On 15-cm plates, C4–12/Flag.ERβ cells were treated with 10 nM E2 for 1 hour then crosslinked with 1% formaldehyde. Cells were lysed with 1 mL of ChIP-lysis buffer (50 mM Tris pH 7.4, 100 mM NaCl, 0.1% SDS, 1% Triton-X, 0.5% NP40, PICIII) and sonicated for 10 cycles, each of which was 10 seconds. The lysate was collected and incubated with 30 uL Protein G Plus-Agarose (Santa Cruz) preimmuned with 10 ug anti-Flag M2 antibodies (Sigma) to capture protein-DNA complex overnight in ChIP-lysis buffer. The Protein G beads were washed once with ChIP-wash buffer I (20 mM Tris pH 8.1, 150 mM NaCl, 0.1% SDS, 1% Triton X, 2 mM EDTA), once with ChIP-wash buffer II (20 mM Tris pH 8.1, 500 mM NaCl, 0.1% SDS, 1% Triton X, 2 mM EDTA), once with ChIP-wash buffer III (10 mM Tris pH 8.1, 250 mM LiCl, 1% NP40, 1% deoxycholate, 1 mM EDTA), and twice with TE buffer. The protein-DNA complexes were eluted with ChIP-elution buffer (100 mM NaHCO3, 1% SDS). The crosslinking was reversed by incubating samples at 65°C overnight. After treatment of RNase A and Proteinase K, the inputs and immunoprecipitated samples were extracted once with phenol/chloroform, once with chloroform, and precipitated in ethanol. The genomic DNA precipitate was suspended in 20 uL nuclease-free water.

### Generation and Characterization of ChIP-seq Libraries

The genomic DNA samples collected from ChIP assays (both inputs and immunoprecipitation products) were processed with Illumina ChIP-seq Sample Preparation Kit. Samples were then sequenced with Illumina Genomie Analyzer II and aligned to hg18 (High-throughput Genomics Analysis Core Facility, University of Chicago, Chicago IL). QuEST [34] was used as the peak-calling software, using default parameters recommended to analyze transcription factor ChIP-seq data. All ChIP-seq data is deposited in the Gene Expression Omnibus (GEO) database at National Center for Biotechnology Information (accession number GSE48161).

The global distribution of ChIP-seq peaks was analyzed using the CEAS package [35]. Distribution around transcription start sites (TSS) was analyzed using in-house algorithms. Enrichment of transcription factor binding motifs was analyzed with CLOVER [36] and the JASPAR public database.

### Global Run-on followed by Sequencing (GRO-seq)

GRO-seq was performed as previously described [32] with limited modifications. Briefly, after 1 hour of E2 or Vehicle treatment, nuclei from C4–12/Flag-ERβ cells were extracted and processed with nuclear run-on assay. The nascent RNA products were ligated to adaptors prior to reverse transcription reaction. The resultant cDNA libraries were then sequenced using Illumina HiSeq2000 (Center for Genome Research and Biocomputing at Oregon State University, Corvallis OR). Sequencing reads were analyzed as described previously [32]. In order to identify E2-regulated genes, we focused on RefSeq-annotated genes, counting reads in a fixed window between +1 kb and +13 kb relative to the transcription start site of each gene, so as to avoid possible complications introduced by paused polymerases [37], and to allow easy side-by-side comparison among samples. The normalized expression value of 1e-5 (normalized against the sample total read counts) was used as the threshold to select genes for further analyses. Comparing E2 treated samples versus vehicle treated samples, genes with FC>1.2 were considered upregulated; those with FC<0.8 were considered downregulated. All GRO-seq data is deposited in the Gene Expression Omnibus (GEO) database at National Center for Biotechnology Information (accession number GSE48161), and the scripts are available upon request.

### Immunoprecipitation

MCF7/C4–12 cells were transfected in 10 cm plates with Flag-ERβ and V5-EBF1 plasmids using FuGENE. Transfected cells were then treated with E2 or Vehicle for 4 hours. Cells were lysed in CoIP-lysis buffer (10 mM Tris pH 7.4, 150 mM NaCl, 0.5% NP40, PICIII) followed by brief sonication. The cell lystate was incubated with 2 ug anti-Flag M2 antibody overnight at 4°C. The complex of interest was precipitated with 30 uL Dynabeads Protein G (Life Technologies) pre-blocked with BSA for 1 hour at 4°C. After the beads were washed, the protein complexes were eluted with SDS loading buffer and then subjected to Western Blot analysis. Three independent experiments was performed. The results of a representative experiment are shown.

### Luciferase Reporter Assay

MCF7/C4–12 cells were transfected in 48-well plates with 3xERE-Luc, pRL-TK, ERα or ERβ, and pcDNA or interactor plasmids using FuGENE. Transfected cells were then treated with E2 or Vehicle for 24 hours. Cells were lysed in Passive Lysis Buffer (Promega) supplemented with PICIII and 1 mM DTT. Lysate was then transferred to 96-well plate for the Dual Luciferase Reporter assays (Promega). Samples and treatments were quadruplicated in each experiment. Statistical analysis was performed using the unpaired Student’s t test; p<0.05 was considered significant. Three independent experiments was performed. The results of a representative experiment are shown as mean ± SD.

### RT-qPCR

C4–12/Flag.ERβ cells were transfected in 6-well plates with pcDNA or EBF1 plasmids using FuGENE (Promega), then treated with E2 or Vehicle for 2 hours. RNA was extracted using Trizol (Life Technologies). cDNA was generated using the Hi Capacity RNA-to-cDNA kit (Life Technologies). qPCR was performed using Fast SYBR Green Master Mix (Life Technologies). GAPDH was used as normalization control. Samples and treatments were triplicated in each experiment. Statistical analysis was performed using the unpaired Student’s t test; p<0.05 was considered significant. Three independent experiments was performed. The results of a representative experiment are shown as mean ± SD.

### Cell Proliferation Assay

C4–12/Flag.ERβ cells were transfected in 24-well plates with pcDNA or EBF1 plasmids using FuGENE (Promega). Cells were treated with E2 or Vehicle for 3 days. Cell confluency, used to quantify cell growth, was measured in an Incucyte FLR live content imaging system (Essen Bioscience). Samples and treatments were triplicated in each experiment. Statistical analysis was performed using the unpaired Student’s t test; p<0.05 was considered significant. Three independent experiments was performed. The results of a representative experiment are shown as mean ± SD.

## Results

### C4–12/Flag.ERβ as a Cell Model to Study ERβ Genomic Functions

Due to the high homology between the DNA-binding domains of the two ERs, ERβ genomic functions should be investigated in a system without ERα interference. For this reason, we used MCF-7.C4–12 cells, a derivative of the MCF-7 breast cancer cell line that no longer expresses any detectable estrogen receptors, as our cell model [31], [33].

To overcome the lack of good ERβ antibodies for ChIP experiments, a Flag-tagged version of ERβ was used, which allowed the specific detection and immunoprecipitation of this nuclear receptor in subsequent experiments.

**Figure 1:**
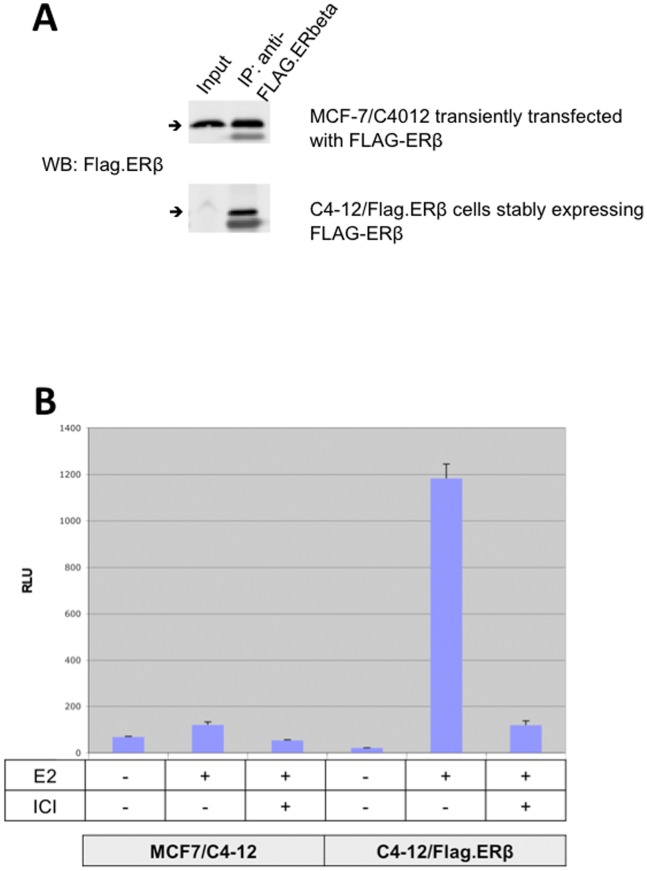
Validation of the C4–12/Flag.ERβ cell line. A – Flag.ERβ stably expressed in C4–12/Flag.ERβ cells was detected (because of low expression level, the ERβ band in the input lane was faint) and immunoprecipitated with anti-Flag antibody. Lysates of MCF-7/C4–12 cells transiently expressing Flag.ERβ were used as positive controls. B – Luciferase reporter assays confirmed the expression of a functional ERβ in C4–12/Flag. ERβ cells. C4–12/Flag.ERβ cells were cultured in 48-well plates, then transfected with 3xERE-Luc and RTK plasmids. After 24 hours of treatment with 10 nM E2 or Vehicle (Ethanol), cells were lysed with Passive Lysis Buffer (Promega) and processed with Dual Luciferase Assay (Promega). (RLU = relative light units; error bars = standard deviation).

As shown in figure 1A, Flag.ERβ is stably expressed in C4–12/Flag.ERβ cells at a low level compared to transiently transfected cells (because of low expression level, the ERβ band in the input lane was faint). The fusion protein could be immunoprecipitated with anti-Flag M2 antibody, confirming the stable expression of ERβ in these cells. An ERE-luciferase reporter assay was used to demonstrate that Flag.ERβ is functional in these cells. As shown in figure 1B, luciferase activity is up regulated upon E2 treatment, indicating the expression of functional ERβ.

In summary, we successfully generated a C4–12/Flag.ERβ cell line in which Flag.ERβ is stably expressed and functional. The tagged receptor was specifically immunoprecipitated with Flag M2 antibody, allowing subsequent experiments to study ERβ genomic functions without interference from ERα.

### Mapping ERβ Genomic Binding Regions in C4–12/Flag.ERβ

**Figure 2:**
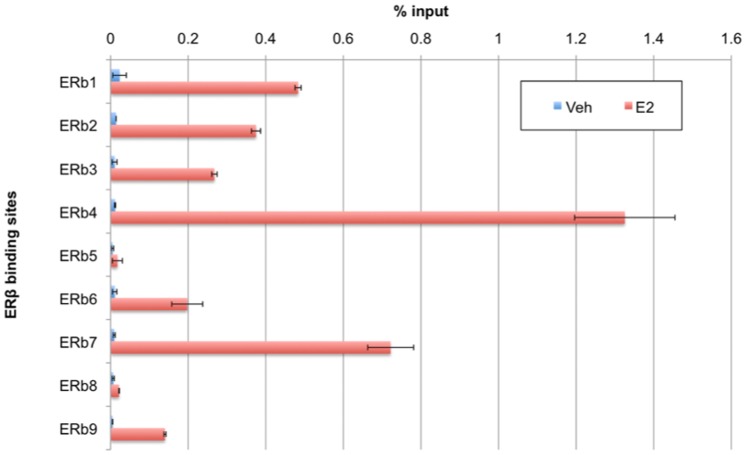
Validation of ERβ binding regions in C4–12/Flag.ERβ cells by ChIP-qPCR. After 1 hour of E2 treatment, C4–12/Flag.ERβ cells were subjected to ChIP assay using anti-Flag M2 antibody. Recruitment of ERβ to representative sites was confirmed with qPCR. (Error bars = standard deviation).

Flag.ERβ was mapped to 3166 binding sites in the C4–12/Flag.ERβ genome by ChIP-seq analysis (1% FDR) after 1 hour of E2 induction. No binding sites were found in vehicle treated cells. Several of these binding sites were chosen at random for validation with ChIP-qPCR. As shown in figure 2, Flag.ERβ is recruited to these genomic loci in an E2-dependent manner.

**Figure 3:**
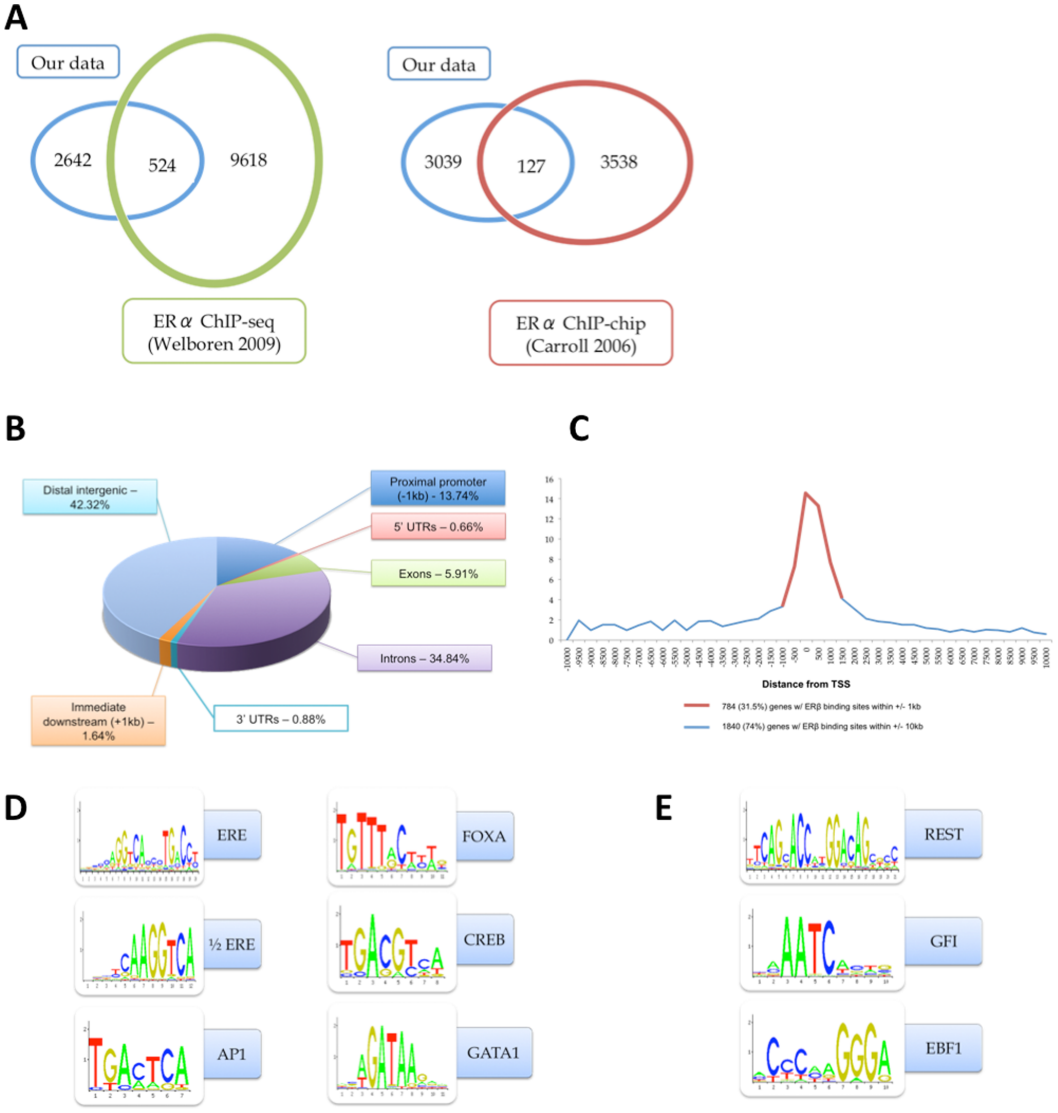
Overview of ERβ binding regions in C4–12/Flag.ERβ cells. ERβ binding sites identified in C4–12/Flag.ERβ cells (blue) were compared with ERα binding sites in MCF-7 cells (green and red). 524 sites are shared with the ERα landscape reported by Welboren 2009 [15]; 127 sites are shared with ERα landscape reported by Carroll 2006 [14]. (B) Global distribution of ERβ: less than 15% of ERβ binding sites were found in proximal promoter regions; most ERβ binding sites were found in introns and distal intergenic regions. (C) Density of ERβ binding sites around gene TSS. (D-E) ERβ binding sites were converted to 400-base long polynucleotides, centered at the binding summit. CLOVER motif analysis [36] was used to identify enrichment of transcription factor binding motifs in this library. JASPAR transcription factor binding motif database was used as the library of known motifs; CpG island, promoter regions and chromosome 20 were used for background analysis. (D) Enrichment of motifs known to associate with ER binding sites. (E) Enrichment of motifs unique to ERβ binding sites in C4–12/Flag.ERβ cells.

As shown in figure 3A, a small percentage of ERβ binding sites overlap with previously reported ERα binding sites in MCF-7 cells [14], [15]. Global distribution analysis showed that ERβ exhibited a pattern similar to ERα in MCF-7 cells: 14% of ERβ binding sites were found at the proximal promoter regions (defined as the regions within 1 kb of any transcription start site); most binding events occurred in intergenic regions or within introns (Figure 3B). ERβ binding sites also exhibited high density surrounding the TSS (figure 3C).

ERβ binding sites were also analyzed for enriched transcription factor binding motifs using CLOVER [36]. The estrogen responsive element (ERE) and half-ERE were found to be the most enriched in our data. AP1, AP2, FOXA, FOXO, CREB and GATA binding motifs were also enriched at ERβ binding sites (figure 3D). Interestingly, we also detected GFI, REST and EBF1 binding motifs, which had not been previously reported to be associated with ERα genomic binding sites (figure 3E).

### Profiling Nascent Transcripts of ERβ Target Genes

**Figure 4:**
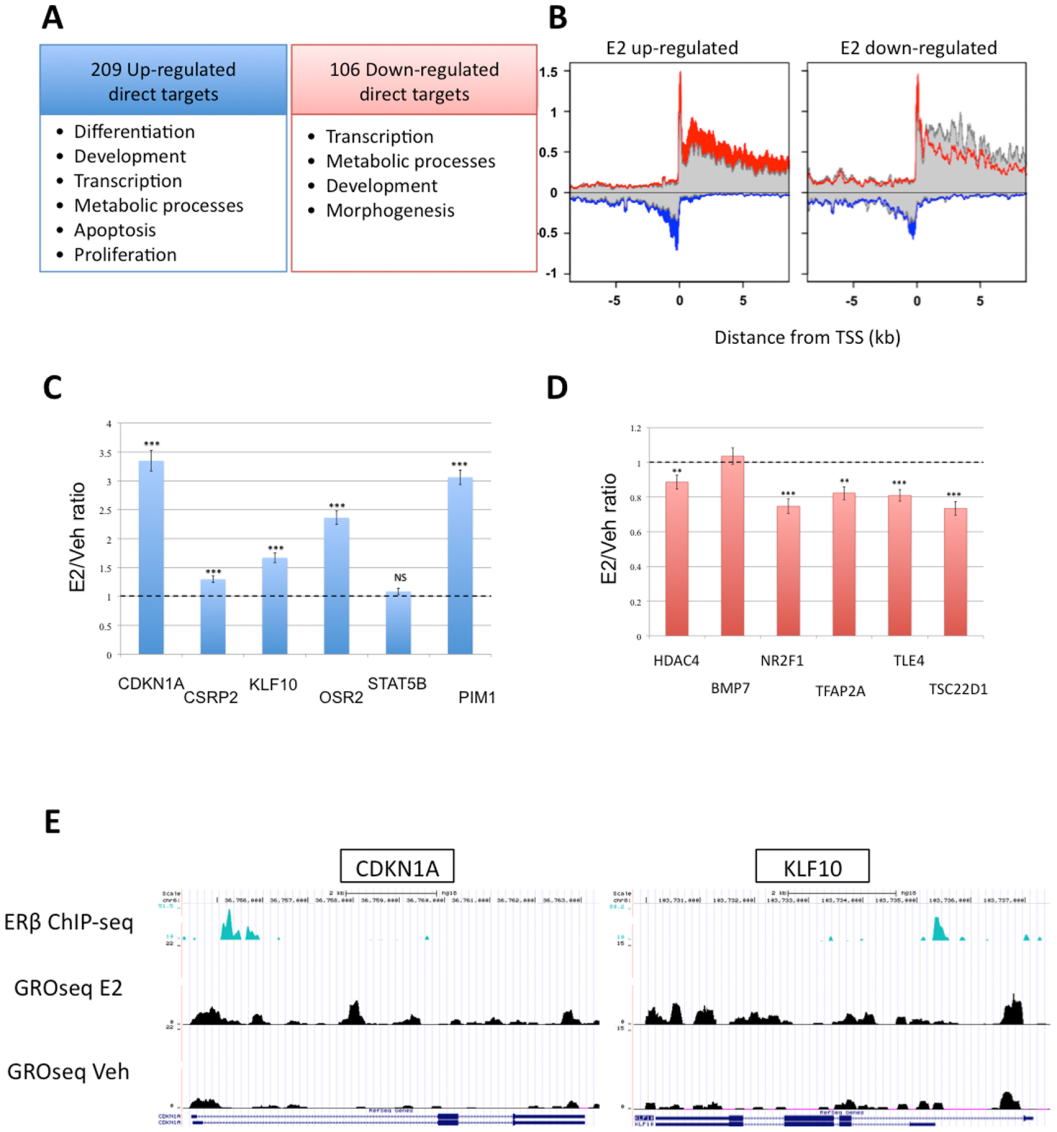
ERβ target genes identified by ChIP-seq and GRO-seq. A – Gene Ontology analysis shows that ERβ target genes are enriched in anti-proliferative processes such as differentiation and apoptosis. B – Metagene anslysis supports genes differentially regulated by ERβ upon E2 treatment. C, D – C4–12/Flag.ERβ cells were treated with E2 for 2 hours, after which mRNA was collected and reverse transcribed to generate cDNA. qPCR focusing on up-regulated (C) and down-regulated (D) genes identified by GRO-seq was used to validate differential gene regulation. (E) ERβ ChIP signal correlated with an increase of GROseq signal at the CDKN1A and KLF10 genes, two representative ERβ targets.

After C4–12/Flag.ERβ cells were treated with E2 for 1 hour (Vehicle treated cells were used as negative control), nascent RNA was collected and profiled by GRO-seq analysis. The list of genes differentially regulated by E2 induction was combined with genes associated with ERβ (defined as those with at least 1 ERβ binding site within −10 kb of TSS and +10 kb of TTS) to generate a list of ERβ target genes. Among 315 ERβ targets, 209 were up regulated and 106 were down regulated (figure 4A). Metagene analysis shows representative views of up- and down-regulated genes (figure 4B).

In order to validate results obtained from our GRO-seq assay, mRNA was collected after 2 hours of E2 induction. A few randomly selected transcripts were analyzed with RT-qPCR. As shown in figure 4C–D, the majority of these transcripts were validated using this approach. Figure 4E shows ERβ ChIP and GROseq signals at CDKN1A and KLF10, two representative ERβ targets. These genes had ERβ binding close to the promoter regions and were upregulated upon E2 treatment (Figure 4C, 4E).

**Figure 5:**
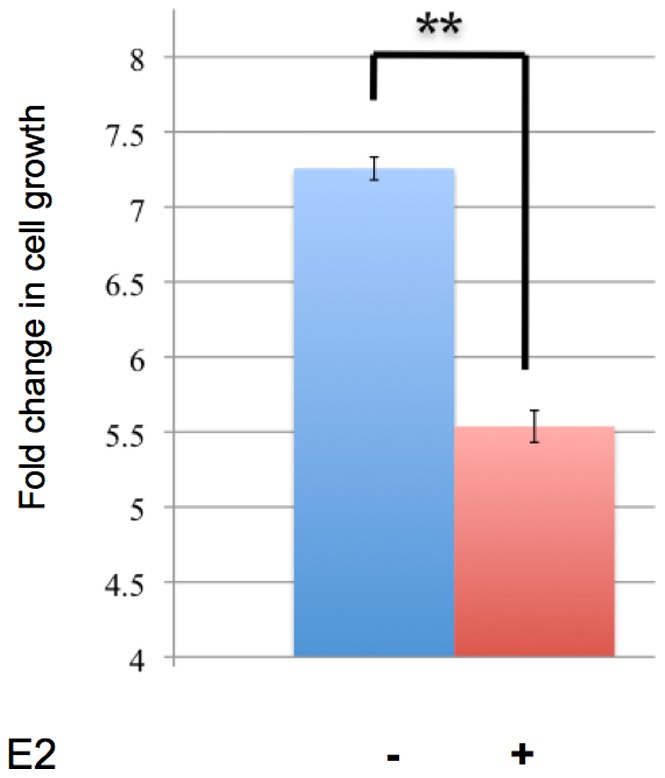
ERβ negatively regulates cell proliferation in an E2 dependent-manner. C4–12/Flag.ERβ cells were treated with 10 nM E2 for 3 days. Cell confluency, detected by the Incucyte FLR live content imaging system (Essen Bioscience), was used to measure cell proliferation. Bar graph represents fold change relative to day 0. (Error bar = standard deviation).

Gene ontology analyses of ERβ target genes revealed specific enrichments in transcription regulation, metabolic processes, differentiation, development and apoptosis (figure 4A). Because the same gene ontology categories were found enriched in both up- and down-regulated groups, there is likely a potential molecular switch in response to E2 via ERβ. The fact that differentiation, development and apoptosis categories were enriched in ERβ target genes suggests that ERβ is anti-proliferative in this cell model. To test this hypothesis, a cell proliferation assay was performed on C4–12/Flag.ERβ cells treated with E2 (or Vehicle as negative control). In agreement with the gene ontology analyses, E2 treatment significantly suppressed C4–12/Flag.ERβ cell proliferation, further supporting the hypothesis that ERβ can function as an anti-proliferative factor (figure 5).

### Association between ERβ and EBF1

**Figure 6:**
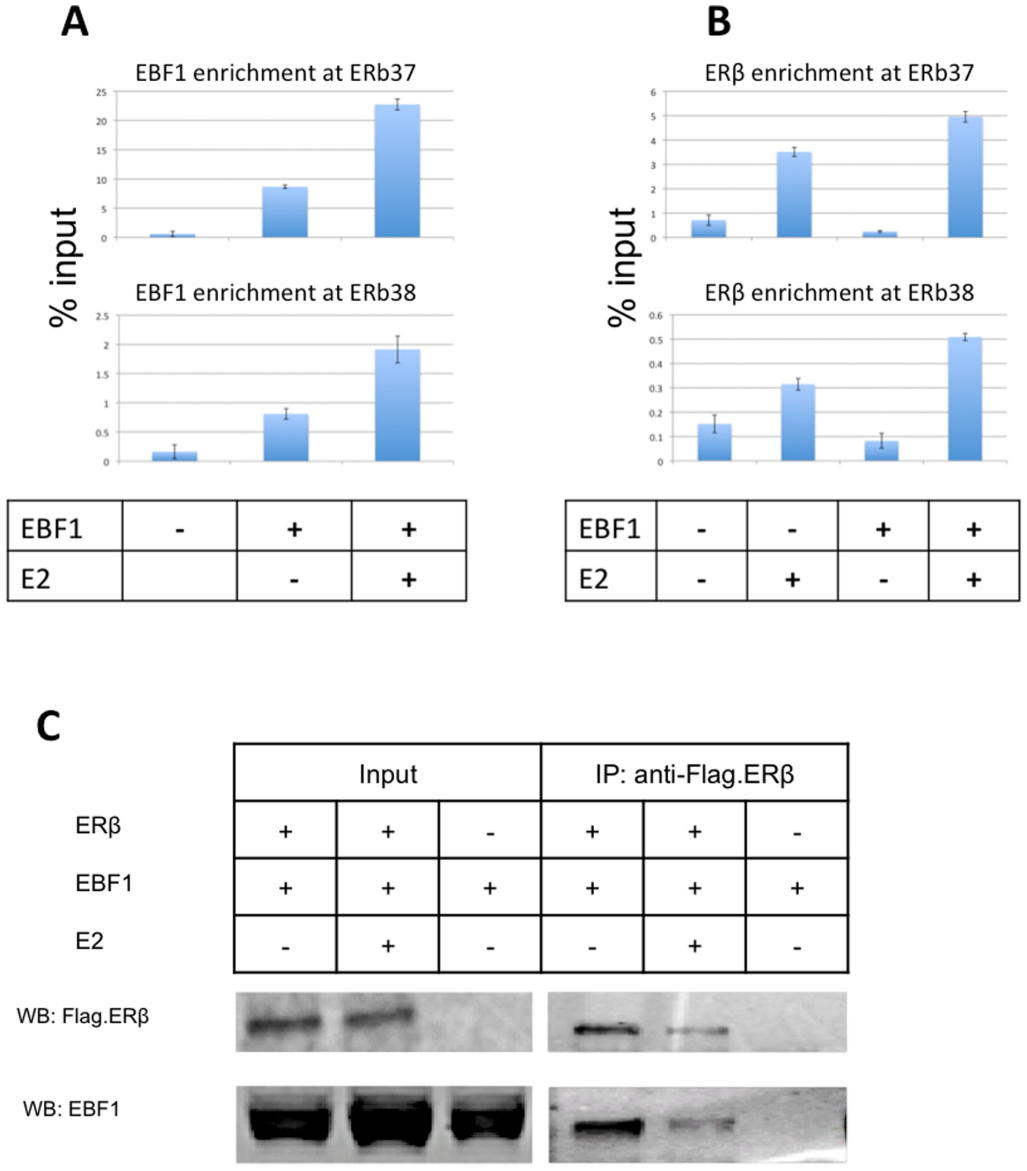
The association between ERβ and EBF1. C4–12/Flag.ERβ cells were transfected withV5-tagged EBF1 and incubated for 1 hr with 10 nM E2. Cells were then treated with 1% formaldehyde and processed by ChIP assay using (A) anti-V5 antibody and (B) anti-Flag M2 antibody. (C) C4–12/Flag.ERβ cells were transfected with V5-tagged EBF1, treated with 10 nM E2, lysed and subjected to co-immunoprecipitation using anti-Flag M2 antibody to immunoprecipitate complexes containing Flag.ERβ. Anti-Flag M2 antibody and anti-V5 antibody were used on Western blots to detect V5.EBF1 and Flag.ERβ, respectively.

Because the EBF1 binding motif was enriched at ERβ binding sites, the interaction between EBF1 protein and a few ERβ binding sites was investigated. When EBF1 was transiently expressed in C4–12/Flag.ERβ cells, it was recruited to several ERβ binding sites in an E2 dependent manner (Figures 6A). Moreover, the recruitment of ERβ at these sites was enhanced in the presence of EBF1 and E2 (figure 6B).

We then used co-immunoprecipitation to test for an interaction between these two proteins. As shown in figure 6C, V5-tagged EBF1 was detected in the ERβ immunoprecipitate, indicating an interaction between the two proteins. However, when V5-EBF1 was immunoprecipitated with V5 antibody, no ERβ was detected (data not shown). These results suggest a stoichiometry in which most of the ERβ interacts with EBF1, while only a small portion of EBF1 interacts with ERβ, indicating that ERβ levels are limiting. Notably, cells used in these assays were treated with MG132 because ERβ levels were very low (figure 6C).

**Figure 7:**
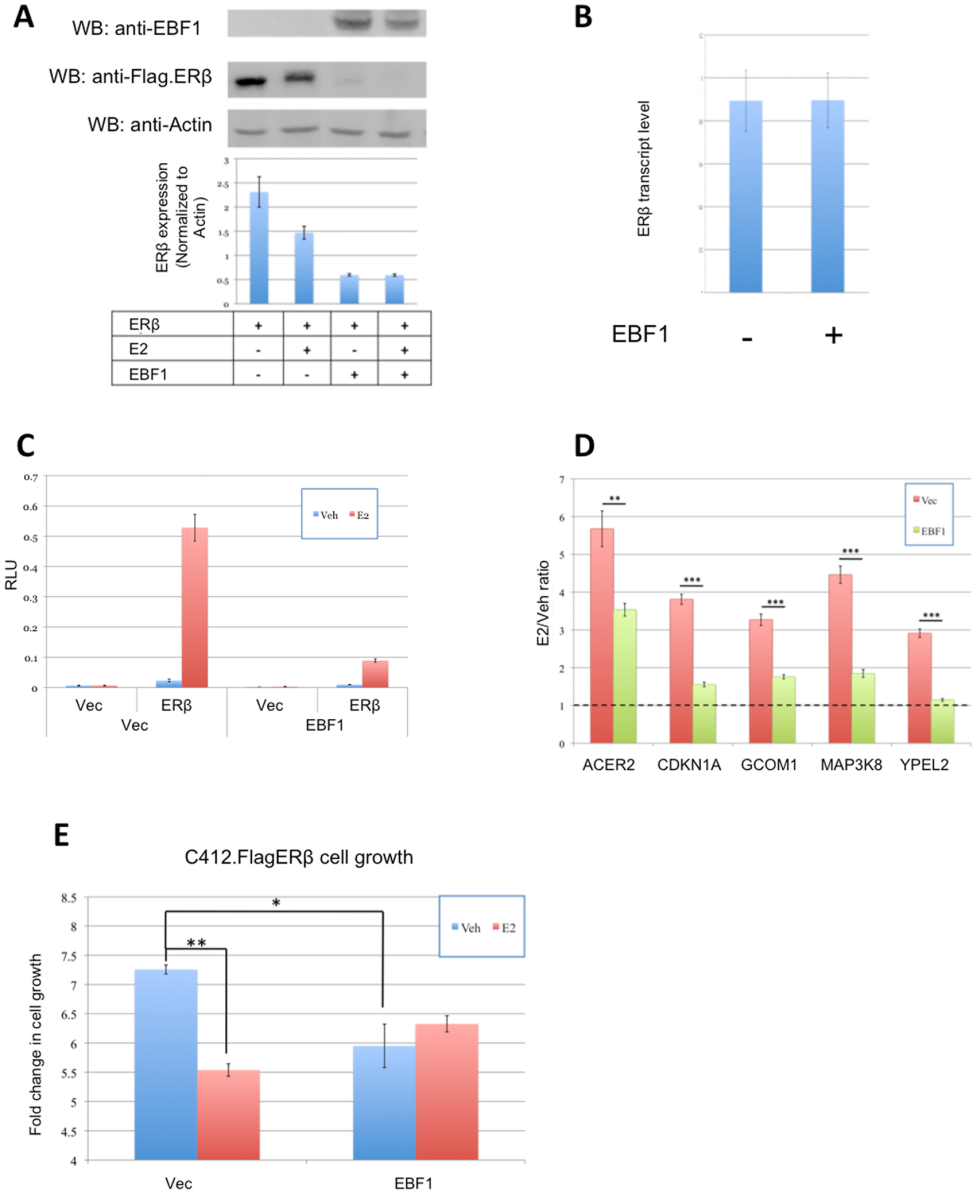
EBF1 negatively regulates ERβ protein levels and downstream activity. MCF-7/C4–12 cells were transfected with Flag.ERβ in the presence or absence of EBF1 and then treated with E2 or Vehicle control. (A) Flag.ERβ protein and (B) mRNA levels were measured on Western blots and by RT-qPCR, respectively. ERβ transcriptional response was measured in an (C) ERE-Luciferase reporter assay as well as (D) by endogenous target gene regulation. (E) C4–12/Flag.ERβ cells were transfected with EBF1 (or pcDNA3.1(-) as Vector control) and treated with E2 for 3 days. Cell confluency, detected by the Incucyte FLR, was used to measure cell proliferation. The bar graph represents the fold change relative to day 0. Error bars represent standard deviation.

Because the presence of EBF1 correlated with low ERβ protein levels, we wished to test the influence of EBF1 on ERβ protein levels and function. Due to the low ERβ expression in stably transfected C4–12/Flag.ERβ cells, transiently over-expressed ERβ was assayed in the presence and absence of EBF1. As shown in figure 7A, EBF1 significantly reduced ERβ protein stability while ERβ transcript levels remained unchanged (Figure 7B), indicating that EBF1 regulates ERβ stability at the protein level. ERβ transcriptional activity was also suppressed, as measured by an ERE-luciferase reporter assay (figure 7C). This suppressive effect was also observed for endogenous target genes. Thus, when C4–12/Flag.ERβ cells were transfected with EBF1, ERβ target gene expression in response to E2 was significantly reduced (figure 7D).

To investigate the role of this cross talk at the phenotypic level, we asked if EBF1 could influence the anti-proliferative effects of ERβ. C4–12/Flag.ERβ cells were transiently transfected with EBF1 and assayed for cell proliferation. As shown in figure 7E, EBF1 significantly suppressed the proliferation of these cells, in agreement with the previously suggested concept that EBF1 is a tumor suppressor [38]. Interestingly, the proliferation of cells transfected with EBF1 became ligand independent.

Altogether, these results confirm that EBF1 negatively affects ERβ protein stability, which in turn down regulates ERβ transcriptional and phenotypic effects.

### EBF1 Differentially Regulates ERα and ERβ

**Figure 8:**
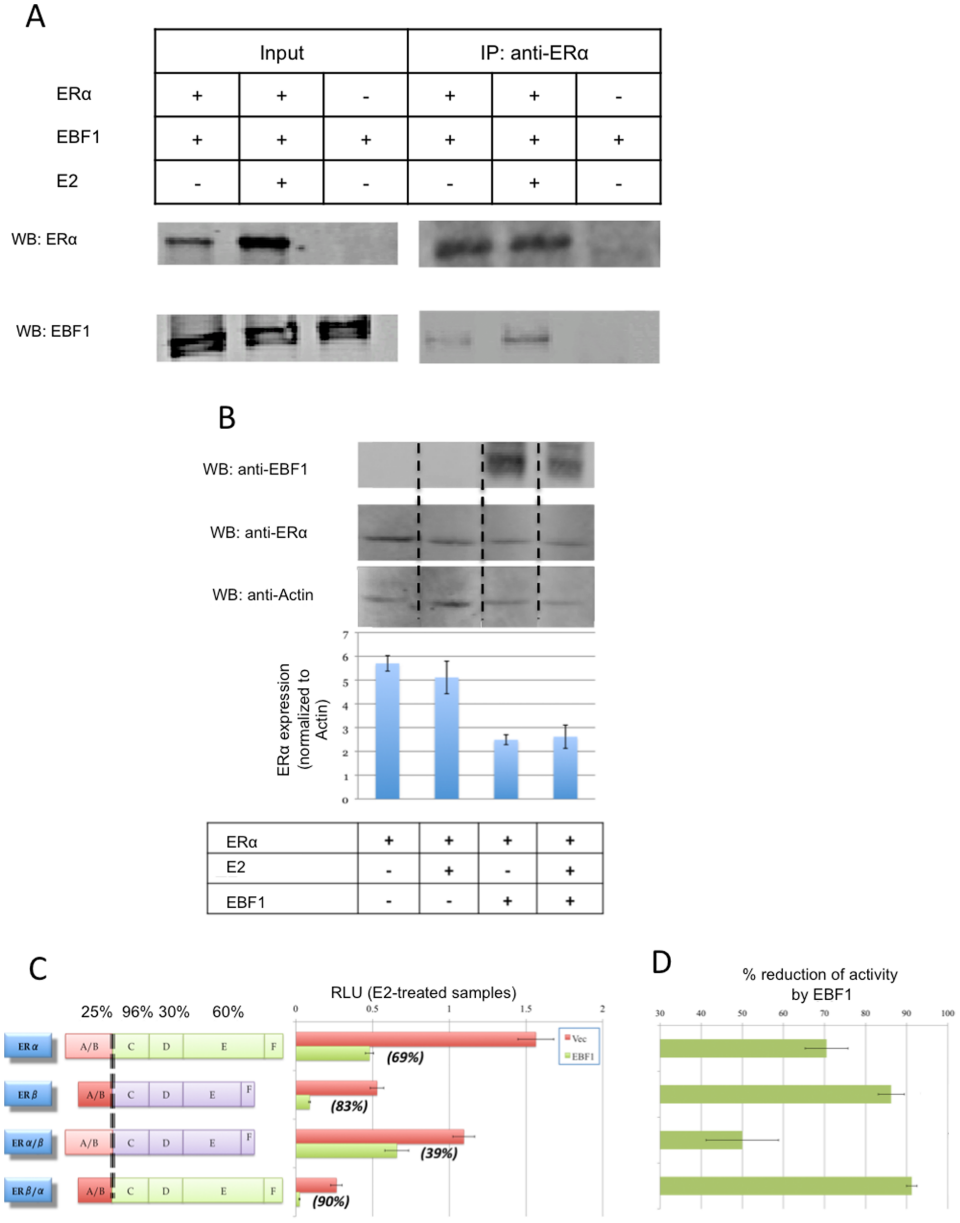
EBF1 differentially regulates ERα and ERβ. (A,B) MCF-7/C4–12 cells were transfected with ERα in the presence or absence of EBF1 and then treated with 10 nM E2 or Vehicle control. (A) Association between ERα and EBF1 was confirmed by co-immunoprecipitation assay. (B) ERα protein levels were measured on Western blots (bands from each row were from the same gel but were rearranged to fit the order similar to that shown in figure 7A on ERβ). (C) Transcriptional activities of different ER constructs were measured with the ERE-Luciferase reporter assay in the presence and absence of EBF1; percentages in parentheses represent how much EBF1 affected each construct, as measured by subtracting luciferase signal in EBF1 transfected samples from their vector controls. (D) Effect of EBF1 on ER transcriptional activities measured by subtracting the luciferase signal in EBF1 transfected samples from their vector controls; bar graph represents the average of three independent experiments; error bars represent standard deviations.

While EBF1-ERβ interaction attenuates ERβ protein stability, it is unclear if this interaction is ERβ specific. We therefore tested the relationship between EBF1 and ERα. In cells transiently transfected with both EBF1 and ERα, as shown in figure 8A, EBF1 is found in the ERα immunoprecipitate, indicating an interaction between the two proteins. This interaction correlates with a decrease in ERα protein levels (Figure 8B). EBF1 also suppresses ERα downstream activity, as measured by reporter assay (figure 8C). Although EBF1 expression affects both ERα and ERβ stability, the suppressive effect was more dramatic on ERβ, indicating a differential regulation of the two estrogen receptors by EBF1.

To further investigate this differential regulation of ERα and ERβ by EBF1, we compared EBF1-mediated attenuation of ERE-luciferase activity of four different ER constructs: full-length ERα, full-length ERβ, a chimeric construct with ERα N-terminal domain followed ERβ C-terminal domain (ERα/β), and a chimeric construct with ERβ N-terminal domain followed by ERα C-terminal domain (ERβ/α) (figure 8C). This approach was chosen, instead of investigating endogenous gene expression levels, so that all samples had the same elements except for those being tested; moreover, variations due to genomic interference were eliminated. Moreover, the identities of common genes being equally regulated by either ERalpha or ERbeta remains elusive and controversial. Therefore, the ERE-Luc reporter assay was an appropriate assay to investigate this cross talk. According to figure 8C, even though EBF1 significantly suppressed the activity of all four constructs, the magnitude of suppression was not the same. In agreement with the observation that EBF1 decreases ERβ protein expression more dramatically than ERα, the transcriptional activity of ERβ was also more reduced in the presence of EBF1 compared to ERα (figure 8C). Interestingly, constructs with ERβ N-terminal domain (full-length ERβ and ERβ/α) were more affected by EBF1 than those with the ERα N-terminal domain. These results indicate that the N-terminal domains of the two receptors are responsible for the differential regulation of ERα and ERβ by EBF1.

## Discussion

Mapping ERα genomic binding sites in MCF7 cells [14] is considered a major milestone in ER research, with significant implications in understanding normal biology and pathophysiology. In particular, the ERα genomic landscape helped identify ERα direct target genes when used in combination with gene expression experiments. Moreover, these findings also revealed distinct ERα binding patterns, which were the basis for further investigations of ERα participation in chromatin looping as well as the identification of ERα binding partners [14], [16], [39]. ERα genomic binding signatures have also been associated with differential clinical outcomes in breast cancer [40].

Our understanding of ERβ function is still very limited compared to that of ERα. Even though the presence of ERβ in diverse tissues and cancers has been demonstrated [12], [41], the expression of this receptor in cultured cell lines remains controversial. This is largely due to the lack of reliable antibodies to detect ERβ protein [4], [23]. These limitations have hindered investigations of ERβ function.

To circumvent these challenges and to study ERβ genomic function, we generated a cell line (C4–12/Flag.ERβ cells) that maintains stable Flag-ERβ expression. Because these cells were derived from the MCF7/C4–12 cells, which no longer express detectable ERs, ERβ functions could be studied without ERα interference. The Flag epitope allowed us to detect and immunoprecipitate ERβ with high efficiency and specificity.

Using this approach, we mapped ERβ to 3166 genomic sites in cells treated with 10 nM E2 for 1 hour. The 45-minute-to-1-hour window of E2 treatment time has been reported to be the first peak of ERα-chromatin binding in MCF7 cells and thus represents ER early transcriptional activities in response to hormone activation [14]–[16], [19], [24]. At this time point, most ERβ binding sites were found in intronic or intergenic regions. Motif analyses of these regions revealed enrichments for many transcription factors that have been shown to interact with ERα. Moreover, motifs that have not previously been associated with ERα binding sites are also enriched, suggesting distinct genomic characteristics of ERβ binding sites.

Interestingly, no ERβ-chromatin binding events were detected in C4–12/Flag.ERβ cells treated with Vehicle (Ethanol). In a study using U2OS cells that express ERβ via a tet-off system (U2OS/ERβ cells), apo ERβ mapped to many genomic loci [25]. The difference between these findings is likely due to the different methods of expressing ERβ. In our stable cell model, ERβ expression is very low and at equilibrium, resulting in no detectable ERβ-chromatin binding in the absence of ligand. The induced, transient expression of ERβ in U2OS/ERβ cells might yield constitutively active ERβ if the amount of expressed protein exceeds the capacity of endogenous chaperones to keep ERβ inactive in the absence of ligand. In addition, the difference between their results and ours could also reflect inherent cell-specific differences between the cell models.

The functionality of ERβ binding sites was addressed by using a GRO-seq assay to profile nascent RNA generated at the time of ERβ-chromatin binding [32]. Conventionally, in order to study the genomic functions of ERs in response to E2, gene expression profiling is performed a few hours after mapping the binding events, allowing mRNA processing to reach completion [14], [19], [24], [25], [32]. Although this approach has been applied to the study of ER target genes, the association between ER binding events and transcriptional regulation is not direct because samples are harvested and measured at different times. Here, we combined ChIP-seq and GRO-seq to map ERβ-chromatin interaction sites and to profile actively transcribed genes at the time of binding events, respectively, to assess the functionality of ERβ binding sites as well as to identify true ERβ target genes.

According to our ChIP-seq results, most of the ERβ binding sites identified in our study do not overlap with ERα sites in MCF7 cells (less than 20%) [14], [15], [19]. This observation indicates that ERβ has a distinct set of target genes, in agreement with previously published studies [16], [19], [24]. According to our GROseq results, in C4–12/Flag.ERβ cells, ERβ target genes are enriched in differentiation, development and apoptosis pathways. Our results are consistent with the observation that ERβ elicits an anti-proliferative effect on MCF-7 C4–12/Flag.ERβ cells in response to E2 stimulation.

It is important to notice that the MCF7 cells and the MCF7/C4–12 cells are not the same due to the lack of ERα expression in the latter. Because ERα is a major transcription factor that regulates a wide array of cellular processes, this difference may lead to differences in cellular physiology [33]. This could contribute to the different genomic landscapes of ERα in MCF7 and ERβ in C4–12/Flag. ERβ cells. However, the MCF7/C4–12 cells were derived from the MCF7 cells, and thus the most related to the MCF7 line. On the other hand, the lack of ERα expression would allow the investigation of ERβ genomic function without the interference from the former receptor. The more desirable comparison would be between ERβ landscape in C4–12/Flag. ERβ cells and ERα landscape in C4–12 cells stably expressing ERα at similar level. However, the investigation of ERα genomic function in such cell line would be beyond the scope of this study. Further studies are required to address this issue.

The global distribution of ERβ sites in C4–12/Flag.ERβ cells is similar to that of ERα in MCF7 cells, with several ERβ-unique features. There are more ERβ binding events in the proximal promoter (>13% versus <7%) and distal intergenic regions (>42% versus 23%) than for ERα in MCF-7 cells [15]. Furthermore, ERβ binding sites exhibit high density proximal to the TSS of target genes, which is not seen for the ERα genomic landscape in MCF-7 cells [14], [24]. Our results are consistent with other studies that have mapped ERβ binding sites in MCF-7 cells expressing ERβ [24], which suggests that this behavior is an ERβ-unique feature. However, it is not yet known whether this distribution pattern reflects endogenous ERβ behavior.

Motif analyses further showed similarities as well as distinct characteristics between ERβ binding sites and those of ERα. Similar to ERα, most ERβ binding sites are enriched in ERE, AP1, AP2, FOXA1, CREB, and GATA motifs, indicating the similarity of ERα and ERβ functional patterns at the genomic level. Similar results have been reported in other ERβ mapping studies [16], [24]. In addition, several transcription factor binding motifs that are not associated with ERα are enriched at ERβ binding sites, including binding motifs for GFI1, REST, and EBF1. These differences between ERα and ERβ genomic landscapes suggest that ERβ target genes likely have different promoter composition and/or structures compared to ERα target genes.

It was interesting to find the EBF1 binding motif enriched in ERβ binding sites. Early B-cell Factor 1 is a crucial transcription factor that drives the maturation of B-cell development. Even though estrogens have been suggested to influence the immune system, an association between EBF1 and estrogen signaling has not been reported. Our finding is the first to suggest a cross talk between EBF1 and ERβ. In addition, the ERE motif was also found in EBF1 binding sites (unpublished data). This association was further validated when EBF1 was co-immunoprecipitated with ERβ. However, ERβ was not detected in EBF1 immunoprecipitated samples, indicating that the stoichiometry of this association was not one to one. While most of the expressed ERβ associated with EBF1, only a small fraction of EBF1 was involved, indicating that ERβ is limiting.

EBF1 over-expression correlated with down regulation of ERβ protein levels. Further experiments revealed that EBF1 negatively regulates both ERs, although the effect was not of the same magnitude for ERα. These observations suggest that EBF1 might be involved in hormone resistant breast cancer. ERα and ERβ have both shared and distinct roles in breast cancer biology, some of which might be antagonistic. One could imagine that EBF-1 functions to differentially modulate the balance between ERα and ERβ activities in breast cancers that express both ER subtypes, resulting in diverse transcriptional and phenotypic consequences.

Additionally, among four ER constructs (ERα, ERβ, ERα/β, and ERβ/α), those carrying the N-terminal domain of ERβ were more sensitive to EBF1, suggesting that the association between EBF1 and both ERs involved the N-terminal region of ER. These results are consistent with the suggestion that the N-terminal region is involved in ERβ degradation [13].

**Figure 9:**
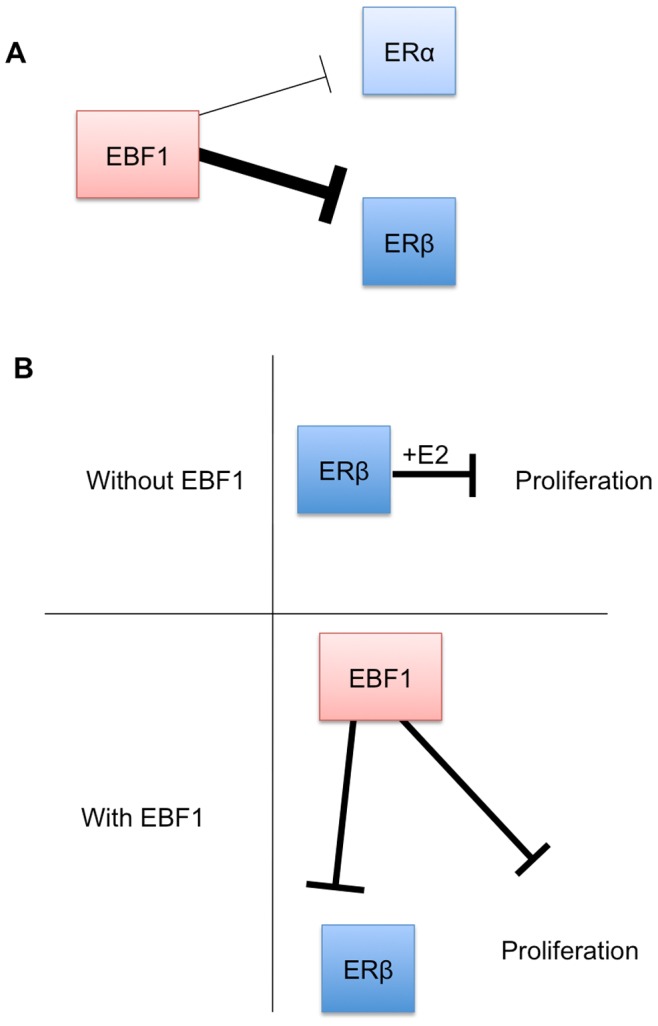
Model of EBF1 antagonistic effects on ERs. A – While EBF1 down regulates both ERs, ERβ is more significantly affected by EBF1. B – In the absence of EBF1, ERβ suppresses cell proliferation upon E2 activation. When EBF1 is present, EBF1 down regulates ERβ levels, rendering the cells hormone insensitive. However, EBF1 also suppresses cell proliferation, independent of ERβ.

Because EBF1 over-expression correlated with the down regulation of ERβ, when EBF1 was transiently expressed in C4–12/Flag.ERβ cells, these cells became insensitive to hormone treatment. EBF1 exogenous expression also suppressed C4–12/Flag.ERβ cell proliferation. This effect is likely to be independent of ER because EBF proteins have been proposed to have anti-proliferative or tumor-suppressive effects [38], [42], Therefore EBF1 over expression may suppress C4–12/Flag.ERβ cell growth independent of its influence on ERβ (figure 9).

Even though our findings are based on engineered cell lines, opposing roles for EBF1 and ER have been reported in normal physiological processes, such as adipogenesis. While ERs suppress adipogenesis, EBF1 promotes the differentiation of adipocytes [7], [38], [43]–[53]. Our observed cross talk between EBF1 and estrogen signaling suggests a connection between the two differentiating mechanisms: EBF1 suppresses the expression and activity of ER, which consequently facilitates adipogenesis by up regulating adipogenic genes as well as by releasing PPARγ from the inhibition exerted by estrogen signaling [51], [52]. Further investigations will be needed to validate this hypothesis.

Another example of the opposing roles EBF1 and ER is found in the differentiation of B cells. EBF1 is a required factor that drives of B cell differentiation to completion. Estrogens, however, inhibit differentiation [7], [54]–[59]. Our results support a possible mechanistic explanation for the opposing roles of EBF1 and ERs in lymphopoiesis. In this model, while EBF1 directly promotes B lymphocyte differentiation, it also mediates the degradation of the two estrogen receptors. By effectively suppressing these potent inhibitors of B cell production, this behavior might represent yet another mode of EBF1 action to promote B cell maturation. Further investigations will be needed to validate this hypothesis.

In summary, with the mapping ERβ genomic binding sites in C4–12/Flag.ERβ cells, we have identified features of ERβ genomic functions that are distinct from those of ERα and from other reported actions of ERβ. In our cell model, ERβ is recruited to and regulates a unique set of genes, some of which suppress cell proliferation. Our analyses also reveal cross talk between ERβ and EBF1. We demonstrate that EBF1 is a negative regulator of both ERα and ERβ. This antagonistic relationship between EBF1 and ERs could explain their opposing roles in different physiological processes. Future experiments will be required to characterize this association in physiologically relevant contexts.
